# Dataset on the psychosocial impact in families with children with developmental and epileptic encephalopathies

**DOI:** 10.1038/s41597-023-02441-3

**Published:** 2023-08-08

**Authors:** Rafael Salom, Luís Miguel Aras, Jessica Piñero, Jon Andoni Duñabeitia

**Affiliations:** 1 Asociación ApoyoDravet, 20009 San Sebastián, Spain; 2 Centro de Investigación Nebrija en Cognición (CINC), Facultad de Lenguas y Educación, Universidad Nebrija, 28248 Madrid, Spain; 3 Servicio Navarro de Salud-Osasunbidea, 31010 Navarra, Spain; 4 Fundación Salud Infantil, 03201 Elche, Spain; 5 AcqVA Aurora Center, Department of Languages and Culture, UiT The Arctic University of Norway, 9019 Tromsø, Norway

**Keywords:** Human behaviour, Human behaviour

## Abstract

Caring for children with developmental and epileptic encephalopathies (DEE) can be challenging for primary caregivers due to the complexity of the condition and the need to provide ongoing care. This has a psychosocial impact on their quality of life, including increased stress, anxiety, and depression, as well as an impact on their support network, work, and relationship with the affected child. It is important that caregivers receive help to manage the psychosocial impact of caring for a child with DEE and promote their long-term well-being. Besides, it is critical that policymakers receive quantitative data about this impact to adequately respond to the needs of these families. To this end, a database was developed using the Childhood Rare Epilepsy Social Impact Assessment (CRESIA) psychosocial impact measurement instrument to quantitatively assess the quality of life of caregivers.

## Background & Summary

This article focuses on families with children with rare diseases, referring to pathologies with a prevalence of less than 5 per 10000 people^1–3^. More than 50% of the rare or low-prevalence diseases related to the central nervous system occur in children and entail social problems for both the children and their families. This is especially critical in neurological rare diseases, which are characterized by high levels of epileptic seizures^4,5^. Among the most serious direct consequences of rare seizure disorders is neurodevelopmental impairment, which impacts on children’s daily and future functioning, causing severe cognitive, physical, and behavioral impairments^6–10^.

The indirect problems arising from the high frequency of developmental and epileptic encephalopathies (DEE) have a major psychosocial impact on the whole family structure^11–13^. The presence of a rare epileptic condition in a child can have a direct and indirect impact on the parents’ quality of life that varies according to different factors such as the severity of the condition, the complexity or restrictions of the symptoms, the coping skills, the social support, and the resources available to cope with its demands^14^. However, one of the most frequent and widespread complaints among primary caregivers is the high psychosocial burden they experience, which is associated with high levels of emotional dysregulation, guilt, anxiety, and stress due to excessive worry and uncertainty about their children’s health status and future^15–17^. This high psychological and physical burden leads in many cases to a decrease in work productivity, social relationships, and economic stability, jeopardizing the quality of life and the integrity of the family system^13,14,18–20^. Epileptic seizures may occur at unexpected times, which may require constant attention and vigilance on the part of families. Besides, the child’s needs may require significant changes in daily routine, such as regular doctor visits, occupational therapy, and physiotherapy. All this can affect parents’ or caregivers’ ability to work, which can lead to financial problems^21–23^.

On this basis, the present article focuses on the creation of a quantitative database of the numerical indicators of the psychosocial impact that primary caregivers of children with DEE. The instrument used to collect the data was the *Childhood Rare Epilepsy Social Impact Assessment* (CRESIA)^24^ scale, which provides quantitative data on caregivers’ quality of life by assessing their social, psychological, general health, family, child-induced stressors, and economic situation. It should be noted that the CRESIA scale has only recently been published and is currently being applied to Spanish speakers, specifically to the Spanish population. The database includes data from primary caregivers of children with DEE, as well as data obtained from a control group of parents with healthy minors. Both groups were included to promote comparative studies with a valid baseline. This database could help other researchers to conduct studies and propose new evidence-based interventions that could improve the well-being and quality of life of all the agents involved in the daily care of a child with DEE.

## Methods

The inclusion criteria for the experimental group required that participating families should (1) be older than 18 years old, (2) speak and understand Spanish as a native language, (3) have a child who has been diagnosed with DEE and has some of the prevalent characteristics associated with DEE, such as intellectual disability, high mortality, motor deterioration and/or behavioral problems for at least 6 months, (4) not having been diagnosed of any mental health disorder in the last 6 months (such as major depression or post-traumatic stress disorder) to ensure that their participation was not affected by mental health problems that could either bias their responses or the interpretation of the research results, and (5) sign the informed consent to participate in the study. The inclusion criteria for the control group were identical except for the fact that the third criterion was substituted for not having a child diagnosed with any known condition. To recruit participants, partnerships were established with associations and foundations specialised in rare diseases related to our research. These collaborations provided access to a wide network of families and individuals affected by these diseases. In addition, social media was actively used as a platform to disseminate information about the study and reach a wider audience. Thus, the recruitment procedure followed was based on a targeted opportunity and snowball sampling method.

The data were collected using the Spanish version of the *Childhood Rare Epilepsy Social Impact Assessment* (CRESIA)^24^ scale and it was presented to family members through the online *Gorilla Experiment Builder* platform^25^ (this tool is subject to the Data Protection Act 2018 and the General Data Protection Regulation). The study evaluated the social, family, health, and psychological areas, the stressors caused by minors, and the economic cost, as well as collected basic sociodemographic data (see Table 1). The items of the instrument were presented and divided into different sections, starting with a brief informed consent form in which the objectives of the study were clearly stated. The study complied with all relevant ethical regulations, and it was approved by the Ethics Committee of Universidad Nebrija (approval code UNNE-2022-006). It should be noted that all individuals participating in this study gave their informed consent for the open publication of the data collected.

**Table 1:** List and type of sociodemographic variables.

Variables	Variable type
Country of origin	Nominal
Gender	Nominal
Age	Continuous
Civil status	Nominal
Education level	Ordinal
Employment situation	Nominal
Economic state	Ordinal
Child gender	Nominal
Child age	Continuous
Diagnosis	Nominal
Age of the child when diagnosis	Continuous
Time between first symptoms and diagnosis	Continuous
Number of seizures of the child per month	Continuous

After providing consent and collecting sociodemographic and clinical information (13 items), they were presented with the CRESIA, which has a total of 371 items. The application time for the entire instrument was approximately 45 minutes. One more item was added to the original CRESIA version to assess the currency used by each participant, given the presence of samples of different Spanish-speaking countries (item number 326). All items were ordered and presented according to the following domains: (a) Social, (b) Health, (c) Psychological, (d) Family, (e) Stressors caused by the child, and (f) Economic. All the continuous items were given and evaluated on a Likert scale in which 1 corresponded to “not at all identified”, 2 to “not very identified”, 3 to “somewhat identified”, 4 to “identified”, and 5 to “very identified”. Participants who were not active in the work market at the time they completed the questionnaire were asked not to respond to the items related to work (items 226 to 250 and 274 to 289). Also, all the items related to rare epileptic diseases were removed from the version of the questionnaire presented to the control group (items 12, 181 to 205, 293, and 302 to 305). The following sections provide a description of each of the domains of the instrument, which can be found in PDF format (Spanish and English version) in Open Science Framework^26^.

### Sociodemographic data

The sociodemographic questionnaire was designed with the aim of collecting specific and personal information from each participant. First, they answered about the country of origin, their gender, age, marital status, employment situation, educational level, and socioeconomic status of the participant. Also, information was collected from the minor in relation to their gender, age, and for the experimental group, the diagnosis of the rare epileptic disease.

### Social

This domain oversees evaluating the social aspect of the lives of families, referring to the social burden that families perceive, as well as their social support and self-concept in society.

### Health

This domain refers to the general physical health of the participants, their self-perception of how they feel or how their health is in general, as well as the physical limitations they suffer due to their child’s rare epileptic disease, and the emotional impact that this physical condition has.

### Psychological

This domain is the most extensively studied one, assessing all psychological areas of the participants that may be affected by the child’s illness. Firstly, it assesses the emotional condition, which is divided into sub-assessments of depressive symptomatology, anxiety, stress, worry, meaning in life, personal growth, and hopelessness. Secondly, it assesses work stress, attending to the work environment, organizational structure, conditions, material work equipment, management, feeling of belonging, and relationships with colleagues, with whom the participant lives. Thirdly, the participant’s self-concept is evaluated, focusing on emotional, intellectual, bodily, and functional self-concept. Finally, the evaluation of the work self-concept is carried out, which involves the participant’s confidence, attitude, and self-image in relation to their work.

### Family

This domain assesses the participant’s family relationships, including the support they receive from their close family environment, as well as the impact of the child’s illness on the family environment.

### Stressors caused by the child

In this domain, an assessment is made of the social, behavioral, emotional, and physiological/biological characteristics of the child that have an impact on the participant’s life.

### Economic

This domain is different from the previous ones described above, as the data are not collected in a Likert-like scale format. These items required multiple choice, dichotomous (yes/no) responses, or open responses in text format (a total of 45 items, plus one additional question on the type of currency used). The aim of this evaluation was to find out the costs of caring for a child with a rare epileptic condition. Therefore, it was divided into three areas of assessment: (a) monthly income: what resources the participant must cope with the financial burden (4 items); (b) direct costs: all expenses directly related to having a child with a rare epileptic seizure disorder (31 items); c) indirect costs: all expenses that are not related to the disorder but are a consequence of it (10 items).

The process of organizing the information of the data collected in the CSV files first required certain pre-processing. As a first step, data from all participants who did not meet the inclusion criteria were eliminated. Next, all the pieces of personal information that could serve to identify the participants were removed to warrant full confidentiality and to adhere to professional secrecy, in strict compliance with Organic Law 3/2018 on the Protection of Personal Data. Third, the presence of any potential duplicate or coding error was evaluated to preserve the integrity of the data presented. And fourth, identifiers for each of the participants, group, and scale were created with the aim of presenting the data with all the necessary information so that it could be analyzed and processed in the best way.

## Data Records

193 adults (154 women) participated in the present study, and the sample was divided into two groups. The first group consisted of a total of 88 adults (67 women), with a mean age of 42.78 years (SD = 6.69). These individuals were parents of minors diagnosed with DEE (47 girls; mean age 9.49 years, SD = 4.89; see Table 2 for the different diagnoses; hereafter, the experimental group). The recruitment process and sample availability resulted in a large percentage (62.5%) of participants being family of children with Dravet syndrome. Despite this bias, 10 additional diagnoses were also included to ensure the generalisability and representativeness of the sample. The second group consisted of a total of 105 adults (87 women), with a mean age of 43.49 years (SD = 6.54). All participants in this group were parents of minors without any diagnosed disease (55 girls; mean age 9.27 years, SD = 5.52; hereafter termed as the control group).

**Table 2:** Different diagnostics of DEE.

Diagnosis	Frequency
Angelman Syndrome	N = 2 (2.27%)
CACNA1A	N = 4 (4.55%)
CDKL5	N = 8 (9.09%)
Dravet Syndrome	N = 55 (62.5%)
GABRA1	N = 1 (1.14%)
Lennox-Gastaut Syndrome	N = 2 (2.27%)
PCDH19	N = 3 (3.41%)
Phelan-McDermid Syndrome	N = 4 (4.55%)
SCN8A	N = 7 (7.95%)
Secondary epilepsy cortical dysplasia	N = 1 (1.14%)
Wolf Hirschhorn Syndrome	N = 1 (1.14%)

The questionnaire CRESIA^24^ used in this study and the extracted data can be found in Open Science Framework (OSF)^26^. The data are provided in raw format in three CSV files, each referring to:

- The sociodemographic data of the experimental and control groups, (file name: ‘Sociodemographic data.csv’). This file presents an organized structure with the following variables divided into columns: Group (experimental or control), Participant identification number (ID), country of origin, gender, age, marital status, educational level, employment status, socio-economic status, and questions related to the child, such as the child’s gender and age, diagnosis of the child, time from first symptom to diagnosis (in months) and the number of seizures the child experienced each month. Data concerning information related to the personal and social characteristics of the two groups are stored here, as well as the personal and social characteristics of the children and the particularities of the diseases they suffer from.
- The data of the different domains and indicators of the CRESIA instrument of the experimental and control groups, (file name: ‘Experimental and control data.csv’). This file presents an organized structure with the following variables divided into columns: Group (experimental or control), Participant identification number (ID), Domain code (A, B, C or D), Domain name (social, health, psychological, family or stressors caused by the child), Indicator code (e.g. A1 and A2 of the domain A. Social), Indicator name (includes the names of the indicators of each domain; e. g. perceived burden, social support and self-concept, of the domain A. Social), Item code (record number to identify the item), Item number (ordinal number of occurrence of the item in the questionnaire), Direct value (the data provided by the sample), and Recoded score (for the items that scores had to be inverted as per the instructions of the questionnaire, the inverted data). Data are stored here concerning information related to the scores according to the domains of the CRESIA instrument: (a) Social, (b) Health, (c) Psychological, (d) Family, (e) Stressors caused by the child.
- Data relating to the scores in the domain f) Economic, only for the experimental group, (file name: ‘Economic domain of the CRESIA.csv’). This file presents an organised structure with the following variables divided into columns: Group (in this case only experimental group), Participant identification number (ID), Domain code (F), Domain name (economic), Indicator code (F1), Indicator name (includes the names of the indicators of each domain; e. g. monthly income, direct costs), Item code (record number to identify item), Item number (ordinal number of occurrence of the item in the questionnaire), and Direct value (the data provided by the sample).

The raw data includes the direct scores as well as the recoded scores for those items that, according to the protocol of the instrument CRESIA, need to be inverted before analysis. Table 3 shows the structure of CRESIA, and descriptive statistics of the results of the parents of minors diagnosed with DEE (Experimental group) and parents of minors without any diagnosed disease (Control group).

**Table 3:** CRESIA structure and descriptive statistics of the results of the parents of minors diagnosed with DEE (Experimental group) and parents of minors without any diagnosed disease (Control group).

Domains and Indicators	Item N	Experimental group				Control group			
		M^a^	SE^b^	SD^c^	Range	M^a^	SE^b^	SD^c^	Range
**A. Social**	43	3.08	0.05	0.47	1.65–3.91	2.28	0.04	0.47	1.52–3.74
A.1. Perceived burden	22	3.29	0.05	0.49	1.95–4.41	2.36	0.05	0.51	1.38–3.67
A.2. Social support and self-concept	21	2.87	0.06	0.61	1.19–4.24	2.20	0.05	0.59	1.24–3.81
**B. Health**	36	2.28	0.06	0.59	1.11–4.17	1.76	0.04	0.50	1.00–3.44
**C. Psychological**	210	2.68	0.05	0.47	1.62–3.77	2.12	0.05	0.52	1.05–3.59
C.1. Emotional condition	146	2.80	0.05	0.53	1.65–3.92	2.20	0.05	0.59	1.22–3.74
C.2. Work stress	25	2.37	0.14	1.26	1.00–5.00	1.69	0.09	1.01	0.00–3.92
C.3. Self-concept	23	2.83	0.06	0.65	1.26–4.30	2.63	0.05	0.59	1.43–4.09
C.4. Work self-concept	16	1.84	0.10	0.88	1.00–5.00	1.44	0.07	0.75	0.00–3.00
**D. Family**	17	2.61	0.05	0.55	1.47–4.53	2.17	0.03	0.31	1.67–3.25
D.1. Perceived family support	6	2.51	0.07	0.65	1.33–4.67	2.46	0.03	0.35	1.60–3.60
D.2. Family satisfaction	6	2.40	0.08	0.82	1.00–4.67	1.53	0.05	0.58	1.00–3.17
D.3. Impact on the family environment	5	2.96	0.08	0.82	1.00–4.80	4.48	0.09	0.95	1.00–5.00
**E. Stressors caused by the child**	19	2.93	0.05	0.55	1.32–4.11	1.71	0.04	0.51	1.00–3.32
E.1. Social manifestations of the child	5	3.46	0.11	1.11	1.00–5.00	1.79	0.07	0.74	1.00–4.80
E.2. Behavioral manifestations of the child	5	2.83	0.09	0.90	1.00–4.40	1.76	0.06	0.68	1.00–4.40
E.3. Emotional manifestations of the child	5	2.68	0.10	1.00	1.00–5.00	1.65	0.05	0.59	1.00–4.00
E.4. Physiological and biological manifestations of the child	4	2.67	0.08	0.78	1.00–4.25	1.64	0.06	0.68	1.00–4.50

***Note:*** ^a^Mean; ^b^Standard Error; ^c^Standard Deviation.

## Technical Validation

All data were collected by experts from three main Spanish institutions: Centro de Investigación Nebrija en Cognición (CINC) from Universidad Nebrija, Asociación ApoyoDravet, and Fundación Salud Infantil. The contact network of these three research, health and third-sector entities facilitated access to many families of children with rare conditions as well as to control families from the same context. The main tool used (the Childhood Rare Epilepsy Social Impact Assessment, CRESIA) is characterized by good internal consistency (with a Cronbach’s Alpha of 0.98). This value of the original tool was validated with an analysis of the internal consistency of the results reported in this dataset, yielding a Cronbach’s Alpha of 0.97 for the experimental group’s data and 0.98 for those of the control group. These results, in addition to the descriptive data characterized by low standard deviations and standard errors of the means per group and domain or indicator (see Table 3), highlight the high degree of homogeneity in the data, reinforcing the validity of the processes of extraction, measurement, and data collection of the generated dataset. It should be noted that the CRESIA scale has only recently been published and is currently being applied to Spanish speakers, specifically to the Spanish population. Due to its recent publication, the CRESIA scale is in the process of expansion. Its external validation and generalization to other languages are necessary so that it can be applied and used in a reliable and valid way in different cultural and linguistic contexts.

## Data Availability

This study didn’t use any customized code.
